# Transcriptional Dynamics of NRF2 Overexpression and KEAP1-NRF2 Inhibitors in Human Cell Line and Primary Lung Cells †

**DOI:** 10.3390/antiox13080924

**Published:** 2024-07-30

**Authors:** Corinne Hamblet, Karin Björhall, Susann Busch, Ulf Gehrmann, Lisa Öberg, Rebekka Kubisch-Dohmen, Sonja Haas, Manish K. Aneja, Johannes Geiger, Carsten Rudolph, Ellinor Hornberg

**Affiliations:** 1 Bioscience Chronic Obstructive Pulmonary Disease & Idiopathic Pulmonary Fibrosis, Respiratory & Immunology, BioPharmaceuticals R&D, AstraZeneca, 431 83 Mölndal, Sweden; 2 Respiratory & Immunology, Neuroscience, Vaccines and Immune Therapies Safety, Clinical Pharmacology & Safety Sciences, R&D, AstraZeneca, 431 83 Mölndal, Sweden; 3 Translational Science and Experimental Medicine Research, Respiratory & Immunology, BioPharmaceuticals R&D, AstraZeneca, 431 83 Mölndal, Sweden; 4 ETHRIS GmbH, 82152 Planegg, Germany; 5 Projects and Early Development, Respiratory & Immunology, BioPharmaceuticals R&D, AstraZeneca, 431 83 Mölndal, Sweden

**Keywords:** antioxidant response, cmRNA, lung, NRF2, KEAP1

## Abstract

Oxidative stress in the human lung is caused by both internal (e.g., inflammation) and external stressors (smoking, pollution, and infection) to drive pathology in a number of lung diseases. Cellular damage caused by oxidative damage is reversed by several pathways, one of which is the antioxidant response. This response is regulated by the transcriptional factor NRF2, which has the ability to regulate the transcription of more than 250 genes. In disease, this balance is overwhelmed, and the cells are unable to return to homeostasis. Several pharmacological approaches aim to improve the antioxidant capacity by inhibiting the interaction of NRF2 with its key cytosolic inhibitor, KEAP1. Here, we evaluate an alternative approach by overexpressing NRF2 from chemically modified RNAs (cmRNAs). Our results demonstrate successful expression of functional NRF2 protein in human cell lines and primary cells. We establish a kinetic transcriptomic profile to compare antioxidant response gene expression after treatment of primary human bronchial epithelial cells with either KEAP1 inhibitors or cmRNAs. The key gene signature is then applied to primary human lung fibroblasts and alveolar macrophages to uncover transcriptional preferences in each cell system. This study provides a foundation for the understanding of NRF2 dynamics in the human lung and provides initial evidence of alternative ways for pharmacological interference.

## 1. Introduction

Reactive oxygen species are important second messengers for many biological processes. When uncontrolled due to chronic cellular stress, these molecules can cause widespread cellular damage in a state called oxidative stress [1]. Oxidative stress is prevalent in diseases of nearly all major organ systems (reviewed [2,3]). One mechanism to combat this stress is the upregulation of the antioxidant response to scavenge free radicals and repair cellular damage. The master transcription factor governing this response is NRF2 (NF-E2-related factor 2). NRF2 is reported to have hundreds of target genes (reviewed [2,4,5]), regulating activities such as glutathione production and recycling, proteohomeostasis, iron metabolism, and lipid detoxification. NRF2 is negatively regulated through its interaction with Kelch-like ECH-associated protein 1 (KEAP1), a cytosolic adaptor protein in complex with the E3-ligase Cul3. KEAP1 interaction leads to constitutive proteasomal degradation of NRF2. Our research group is interested in lung diseases, such as chronic pulmonary obstructive disease, where oxidative stress is widely reported, but the levels of NRF2 and its target genes vary between reports [6,7,8,9,10,11,12,13,14].

**Figure 1:**
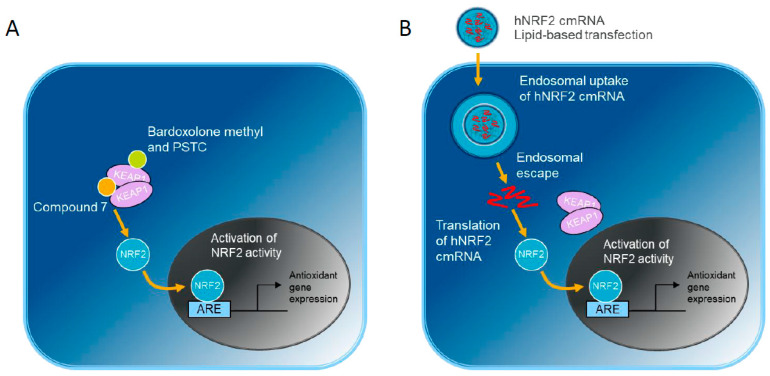
NRF2 activation strategies compared in this study: (**A**) Small molecules targeting KEAP1 inhibit NRF2-KEAP1 interaction. (**B**) Alternatively, hNRF2 cmRNA transfection results in endosomal uptake of the cmRNA-lipid nanoparticle. Following endosomal escape, the hNRF2 cmRNA is translated into NRF2 protein. Both pathways culminate with NRF2 translocation into the nucleus and upregulation of antioxidant gene expression.

Pharmacological upregulation of the antioxidant response is a target for the treatment of oxidative stress-driven diseases (Figure 1A). One strategy has been to interrupt the KEAP1-NRF2 interaction directly with small molecule protein–protein interaction inhibitors, such as Compound **7**, which sits in the Kelch domain binding pocket [15]. Other small molecules, such as Bardoxolone methyl [16,17] and 3-(Pyridin-3-Ylsulfonyl)-5-(Trifluoromethyl)-2H-Chromen-2-One (PSTC, [18]) target the cysteine residues in the Broad-Complex, Tramtrack and Bric-a-brac (BTB) domain resulting in NRF2 liberation. However, these modes of activation could have effects beyond the modulation of NRF2 levels. In addition to NRF2, the Kelch domain binding site on KEAP1 is known to interact with a growing number of non-canonical cargoes, which may further influence downstream signaling of the NRF2-dependent and -independent pathways (reviewed [19,20]).

We investigated an alternative approach by overexpressing human NRF2 (hNRF2) to determine the effects of NRF2 modulation alone (Figure 1B). In this system, in vitro transcribed hNRF2 mRNAs are delivered to cells. The introduction of chemically modified nucleotides reduces immunogenicity and intracellular degradation [21,22,23,24,25]. Delivery is achieved through lipid-based transfection. Following endosomal escape, hNRF2 mRNA can be translated above endogenous levels to activate the antioxidant pathway. We first evaluated the expression and function of the constructs in a lung epithelial cell line. Next, we performed functional experiments in more relevant pulmonary primary cell systems: epithelial cells, fibroblasts, and alveolar macrophages.

## 2. Materials and Methods

### 2.1. Cultivation of Commercial Cells

BEAS-2B cells (ATCC, Manassas, VA, USA) were cultivated in LHC-9 medium (Thermo Fisher Scientific, Waltham, MA, USA) in coated flasks (0.01 mg/mL BSA, 0.03 mg/mL Collagen type I, 0.01 mg/mL fibronectin). ARE-HepG2 cells (BPS Bioscience, San Diego, CA, USA) were cultivated in minimal essential media (MEM) supplemented with 10% fetal bovine serum (FBS), 1% non-essential amino acids, 1 mM sodium pyruvate, 1% penicillin/streptomycin (P/S), and 0.6 mg/mL geneticin. The same medium without geneticin was used for thawing. Human pulmonary fibroblast (HPF) cells (PromoCell, Heidelberg, Germany) were cultivated in D-MEM supplemented with 10% FBS. Sub-cultivation was performed using 0.05% trypsin. After trypsinization, trypsin was deactivated using a trypsin inhibitor (BEAS-2B) or cultivation medium (ARE-HepG2 and HPF). Therefore, an equal or greater volume of trypsin inhibitor/culture medium as trypsin was used.

Normal human bronchial epithelial cells (HBECs), isolated from the surface epithelium of human bronchi of 6 different donors, were purchased from Lonza (Basel, Switzerland). Passage 3 cells were expanded in the PneumaCultTM-Ex Plus medium (STEMCELL™ Technologies, Vancouver, BC, Canada). Cells were dissociated from culture flasks with StemPro Accutase (Thermo Fisher Scientific) and seeded into 96-well cell culture plates at a density of 15,000 cells/well for transfection.

All cells were cultivated at 37 °C in a humidified atmosphere with 5% CO_2_.

### 2.2. mRNA Transfection and Compound ***7*** Treatment

Compound **7** (shortened to C7 in some figures) was synthesized at Pharmaron (Beijing, China). PSTC was synthesized at AstraZeneca AB (Gothenburg, Sweden). Bardoxolone methyl was purchased from CarboSynth (Compton, UK). hNRF2 cmRNAs were synthesized at Ethris GmbH by in vitro transcription. Cells were seeded in their respective density 24 h before treatment. Fresh medium was added to each well (100 μL per 96-well, 200 μL per 48-well) before transfection. All mRNAs were transfected using Lipofectamine^®^ MessengerMAX™ (Thermo Fisher Scientific) in an RNA-to-Lipofectamine ratio of 1:1.5 (*w*/*v*). Cells were treated with 100 μL Compound **7** or 125 μL final volume of mRNA nanoparticles. For the 48 h treatment of BEAS2B and HPF cultures, a medium change was conducted after 24 h of incubation with 100 μL fresh cultivation medium without mRNA/Compound **7**.

### 2.3. Antioxidant Response Element (ARE) Luciferase Assay

ARE-HepG2 cells were seeded in a 96-well plate 24 h before treatment. For the induction of ARE, cells were treated with different concentrations of wildtype and mutant NRF2-mRNA and Compound **7**. After treatment, the supernatant was removed from the plate, the cells were washed once with phosphate-buffered solution (PBS), and the plate was frozen at −80 °C until luciferase activity measurement. For luciferase measurement, plates were thawed and lysed with 100 μL lysis buffer (0.25 M TRIS, 1% Triton X-100, pH 7.8). To complete lysis, plates were incubated for 20 min on a shaker (400–600 rpm) at room temperature. Afterward, plates were placed on ice, and 50 μL cell lysate from each well was transferred into a 96-well white flat bottom microplate. Luciferase activity was measured using a Tecan Infinite M200 Reader device (Männedorf, Switzerland) by dispensing 100 μL of the luciferase assay buffer (0.47 mM D-luciferin free acid, Synchem, Felsberg, Germany) automatically and the measurement was the resulting chemiluminescence. For all statistical analyses, the comparison is to the relevant control (for cmRNAs, the hNRF2-STOP or eGFP controls; for small molecules, the dimethyl sulfoxide–DMSO control).

### 2.4. Western Blot

For cell lysis, cells were washed with 200 μL cold PBS to remove any residual traces of the medium and frozen at −80 °C. To ensure complete lysis, the cells were lysed directly in the plate by adding 60 μL/well M-PER lysis buffer (Thermo Fisher Scientific) complemented with complete, EDTA-free protease inhibitor (Roche, Basel, Switzerland), and 40 μL/mL DNase I solution (Thermo Fisher Scientific) on ice for 30 min before Western blot was performed. Samples were mixed with LDS Sample Buffer and Reducing Agent and heated for 10 min at 70 °C. Gel electrophoresis was performed using NuPAGE™ 4–12% Bis-Tris Midi Gels (Thermo Fisher Scientific). Separation was performed in a NuPAGE™ Midi Gel Tank (Thermo Fisher Scientific) for 40 min at 200 V. Separated proteins were blotted using the TransBlot^®^ Turbo™ Transfer System for 30 min using Turbo Transfer Pack Midi 0.2 μm PVDF (Bio-Rad, Feldkirchen, Germany). After the transfer, membranes were blocked with NET-gelatin (0.25% *w*/*v*) at room temperature (RT) for 1 h. The membrane was incubated with rabbit anti-NRF2 (EP1808Y, abcam ab62352, Cambridge, UK) and anti-beta actin (SP124, abcam ab8227, Cambridge, UK) for housekeeping control, both mixed together in NET-gelatin. Primary antibodies were incubated overnight at 4 °C. After three washes of 10 min each at room temperature with NET-gelatin, horseradish peroxidase (HRP)-conjugated secondary antibody diluted (goat anti-rabbit IgG-HRP, abcam ab205718, Cambridge, UK) was added at RT for 1 h. Membranes were washed again three times 10 min each at RT with NET-gelatin and incubated with a chemiluminescent substrate following the manufacturer’s instructions (Luminata Crescendo Western HRP substrate or Luminata Classico, Merck KGaA, Darmstadt, Germany). Signals were quantified using the ChemiDoc™ MP System (Bio-Rad), and relative NRF2 abundance was calculated against relative actin abundance. For all statistical analyses, the comparison is to the relevant control (for cmRNAs, the hNRF2-STOP or eGFP controls; for small molecules, the DMSO control).

### 2.5. RT-qPCR (BEAS-2B and HPF)

Cells were lysed using the SingleShot™ Cell Lysis Kit (Bio-Rad). For cell lysis, the cell culture medium was aspirated, and cells were washed twice with 300 μL D-PBS per well before the plates were frozen at −80 °C. An amount of 50 μL lysis buffer for RNA isolation was added per well and incubated for 10 min at RT. Subsequently, the cell lysate was transferred to a PCR plate. Protein and DNA were digested by incubation for 5 min at 37 °C followed by an incubation for 5 min at 75 °C. cDNA Synthesis was performed using iScript™ Select cDNA Synthesis Kit (Bio-Rad). The plates with the cell lysates were thawed on ice. All kit components, except iScript™ reverse transcriptase, were thawed on ice, mixed thoroughly, and centrifuged briefly. cDNA synthesis was performed using Oligo (dT) primers. An amount of 4 μL of the cell lysate was pipetted in a new PCR plate using a multi-channel pipet before adding 16 μL of the master mix on top. The plate was sealed using a cover foil and mixed gently at 400 rpm before the plate was spun down briefly. cDNA synthesis was performed using a thermal cycle. Universal probe library (Roche) was used for RT-qPCR to detect *HMOX1*, *SRXN1*, *TXRND1*, *NQO1,* and *GCLM*. *ActB*, *HPRT1,* and *RPLP0* were used as reference genes. Sequences are listed in Supplemental Table S1. cDNA was thawed on ice, and 2 μL of cDNA was mixed with 2 μL RNAse-free water and pipetted in a LightCycler^®^ 480 Multiwell Plate 96. Therefore, all components of the FastStart Essential DNA Probes kits were thawed. Components were mixed thoroughly, briefly spun down, and stored on ice. The master mix was added to the cDNA using a multi-pipette. The plate was covered with LightCycler^®^ 480 Sealing Foil and spun down briefly. qPCR was performed using LightCycler^®^ 96 System (Roche). For all statistical analyses, the comparison is to the relevant control (for cmRNAs, the hNRF2-STOP or eGFP controls; for small molecules, the DMSO control).

### 2.6. Human Cytokine Measurement (IL-8)

For the determination of immunogenic potential, cytokines were analyzed in the supernatants of transfected cells. At 24 h after transfection, 110 μL supernatant from HPF cells was transferred to a pre-cooled new 96-well plate (flat bottom, TPP), and the plate was immediately frozen at −80 °C. Cytokines were analyzed using Human IL-8/*CXCL8* XL Magnetic Luminex Performance Assay (Biotechne, Minneapolis, MN, USA). The measurement procedure was performed as stated in the manufacturer protocol. Standards were measured in duplicates.

### 2.7. HBEC Next-Generation Sequencing (NGS) Study Sample Generation

Cells were transfected or incubated with small molecule compounds, then the media was removed, and lysis buffer plus Proteinase K (Agencourt RNAdvance Cell v2) was added at the indicated timepoints. After 30 min at room temperature, cell lysates were stored at −80 °C until RNA extraction. RNA extraction was performed using magnetic bead-based technology by RNAdvance Cell for total RNA isolation (Beckman Coulter Life Sciences, Brea, CA, USA) in a Biomek workstation (Beckman Coulter Life Sciences). Integrity and quantity of RNA were assessed by Fragment Analyzer Standard Sensitivity RNA kit (Agilent Technologies, Santa Clara, CA, USA). All samples had an RNA integrity number of 10 and were deemed of sufficient quality and quality for mRNA-seq analysis.

### 2.8. NGS Study-Library Preparation, Sequencing, and Data Clean-Up

NEBNext Ultra II Directional RNA Library Preparation Kit was used to build libraries for 240 samples from which high-quality RNA (RQN 9.5–10) had been obtained. Five samples failed library prep. The remaining 235 libraries that passed quality control (QC) were sequenced using a paired-end sequencing approach (2 × 150 bp) on an Illumina NovaSeq platform to an average depth of 24 million read pairs; all met the QC (≥80% of bases ≥ Q30). Library prep and sequencing were conducted by GeneWiz, Leipzig, Germany, and raw sequencing data (fastq files) were delivered to AstraZeneca.

Raw sequence data (fastq files) generated for the 235 successful libraries were processed. Ribosomal reads were filtered with RiboDetector (v.0.2.6), and sequencing adapters were trimmed using NGmerge (v.0.3). Read quality for all libraries was accessed using FastQC (v.0.11.9), Qualimap (v.2.2.2d) and samtools stats (v.1.15). QC metrics for Qualimap were based on a STAR (v.2.7.10a) alignment against the human genome (GRCh38, Gencode v43). Next, QC metrics were summarized using MultiQC (v.1.12). A human transcriptome index consisting of entries from Gencode (v43) was generated, and reads were mapped to the index using Salmon (v.1.7.0). The bioinformatics workflow was organized using Nextflow workflow management system (v.20.10) and Bioconda software management tool.

Sequence data from one sample did not pass QC as it had a strong and deviating 3′bias and was also an outlier in the principal component analysis (PCA) plot and was hence excluded. Furthermore, all 6 h timepoint samples for two of the donors (N3643, N8571) were excluded from the downstream analysis based on an odd expression profile of gender-specific genes for which no logical explanation could be found. Thus, expression data from 218 of the 240 samples passed QC and were included in further analysis.

### 2.9. Differential Gene Expression Determination in NGS Study

Differential gene expression was assessed with DESeq2 (v.1.34.0), using ashr (v2.2_54) for fold change shrinkage and the Benjamin Hochberg method for multiple testing correction. Estimated counts from Salmon were used as input for DESeq2 using tximport (v.1.22.0). Differential expression was assessed between different treatment groups within each timepoint, including donor as a factor in the model to account for donor effects. An adjusted *p*-value of <0.05 was considered significant, and a log2foldchange cut-off of 1 was also applied. Transcripts were excluded for future analysis when they did not have a valid gene symbol assigned. Protein-coding transcripts were defined by gene biotype indexing in Gencode (v43). Data were analyzed through the use of Ingenuity Pathway Analysis [26] (IPA, Qiagen, Hilden, Germany).

### 2.10. Alveolar Macrophages

Alveolar macrophages were isolated from donors (*n* = 6) undergoing tissue resection for non-malignant lung cancer. The study was conducted according to the guidelines of the Declaration of Helsinki and approved by Etikprövningsnämnden (Dnr 2021-00086) in Gothenburg, Sweden, and informed consent was received from all study subjects.

Tissue was placed in DMEM with Glutamax, 10% FBS, non-essential amino acids, and P/S (all purchased from Gibco, Thermo Fisher Scientific) for transport before being placed in a large petri dish for cell isolation. Using a 19G needle, sterile PBS without Ca++/Mg++ was injected at random sites into the tissue up to 10 times or until PBS flushes looked clear. PBS was collected in 50 mL Falcon tubes and centrifuged 300× *g* for 5 min at RT. After carefully removing the cell-free supernatant, cells were resuspended in pre-warmed X-Vivo15 media (Lonza) supplemented with 2 mM L-Glutamine and 1X PEST. Cells were counted and plated in 96-well plates at approximately 160,000 viable cells/well before being incubated at 37 °C and 5% CO_2_ for 1 h. The medium was vacuum-aspirated from each well, and 100 µL media was added up to 5 times to wash away erythrocytes. After the final wash, 180 µL media was added, and cells were rested for 2 h at 37 °C and 5% CO_2_. Wells were washed twice with 200 µL PBS to remove non-adherent cells. Cells were then transfected or incubated with small molecules in X-vivo 15 (Lonza) for up to 48 h. The transfection conditions used are shown in Table 1 and follow the protocol for Lipofectamine MessengerMax (Invitrogen, Carlsbad, CA, USA, Thermo Fisher). 

At the end of the culture, cells were lysed in RLT buffer, and RNA was prepared according to the manufacturer’s instructions (Qiagen). qPCR was performed as described above. Taqman assays used are in Supplemental Table S2.

## 3. Results

### 3.1. Expression of Functional NRF2 in Human Cell Lines

To select the most efficient human NRF2-encoding, chemically modified mRNA (hNRF2 cmRNA) for this study, four mRNA sequences were assessed for expression and activity in cell lines. Two different exogenous 5′-untranslated region (UTR) sequences, *human alpha-globin* (*hAg*) and *minimal* (*min*), were tested in combination with either the wildtype (wt) human NRF2 coding sequence or an E79K mutant form. This mutation reportedly allows NRF2 protein to bypass KEAP1 interaction [27]. In contrast, wt NRF2 protein is considered to be subject to KEAP1 binding and, therefore, constitutive degradation.

**Figure 2:**
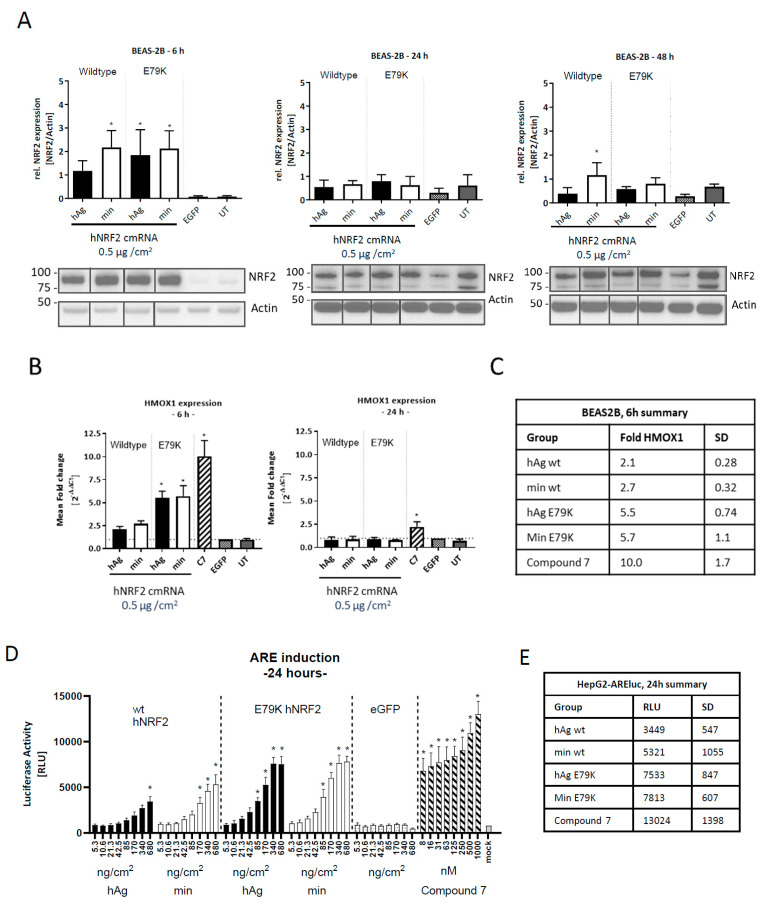
Functional NRF2 expression from hNRF2 cmRNA in human cell lines. (**A**) NRF2 protein expression following transfection of hNRF2 cmRNAs in BEAS-2B cells. The full Western blot images were cropped to the relevant NRF2 band. (**B**) HMOX1 gene expression following transfection of hNRF2 cmRNAs in BEAS-2B cells. Data are pooled results from 3 independent experiments showing mean ± SD. * *p* < 0.05; ordinary one-way ANOVA with Dunnett’s multiple comparisons against eGFP-transfected control at each timepoint. (**C**) Table detailing mean values from (**B**) and SD. (**D**) ARE-luciferase values 24 h after transfection of hNRF2 cmRNAs in ARE-luc HepG2 cells. * *p* < 0.05; ordinary one-way ANOVA with Dunnett’s multiple comparisons against 5.3 ng/cm^2^ eGFP-transfected control. (**E**) Table detailing mean maximum luciferase signal for each treatment group in (**D**) and SD.

To test the expression and kinetics of NRF2 protein translation, a lung epithelial cell line (BEAS-2B) was transfected with the construct, and NRF2 protein expression was assessed between 6 and 48 h (Figure 2A). At 6 h after transfection, NRF2 expression increased for wt NRF2 with the min 5′ UTR as well as for mutant protein with both 5′ UTR sequences compared to eGFP-transfected control. At 24 and 48 h after transfection, NRF2 expression levels were equivalent to untransfected endogenous levels.

The functionality of the overexpressed NRF2 was assessed by measuring the upregulation of the NRF2 target gene heme-oxygenase 1 (*HMOX1*). Maximal *HMOX1* transcript upregulation was observed in BEAS-2Bs at 6 h post-transfection, and by 24 h, it had returned to baseline for all hNRF2 cmRNAs (Figure 2B). Despite similar NRF2 protein expression between the hNRF2 wt and hNRF2 E79K cmRNAs at 6 h, the E79K mutant resulted in higher *HMOX1* upregulation (5.5- to 5.7-fold compared with 2.1- to 2.7-fold). NRF2 protein translated from all hNRF2 cmRNAs induced lower *HMOX1* than the KEAP1 small molecule inhibitor at the chosen doses and timepoints, Compound **7**, which upregulated *HMOX1* 10.0-fold at the 6 h timepoint and 2.2-fold at 24 h (Figure 2C).

To assess whether these observations were specific to *HMOX1* gene expression or more broadly applicable to all NRF2 target genes, hNRF2 cmRNAs were transfected into a cell line (HepG2) containing an ARE-luciferase reporter gene. A dose-dependent increase in activity was observed with all hNRF2 cmRNAs and Compound **7** (Figure 2D). Compound **7** treatment resulted in significant luciferase expression at all dose levels tested (Figure 2E). For all remaining studies, the hNRF2-cmRNA with the *minimal* 5′ UTR was used.

### 3.2. Identification of an NRF2 Gene Signature in Primary Human Bronchial Epithelial Cells

To investigate the effect of NRF2 expression on global gene expression in primary cells, hNRF2 cmRNAs were transfected into human bronchial epithelial cells (HBECs). Upregulation of *HMOX1* was observed at 24 h with both hNRF2 cmRNAs (Supplemental Figure S1A). The small molecule KEAP1 inhibitors Compound **7**, Bardoxolone methyl, and PSTC were also evaluated for this study and induced *HMOX1* gene expression at 24 h (Supplemental Figure S1B).

Next, a transcriptomics study was run in order to compare whether NRF2 overexpression by cmRNA transfection has similar downstream effects on global target gene expression as KEAP1 inhibition by small molecules. EC80 doses of all compounds were established using HMOX1 gene expression at 24 h in submerged human bronchial epithelial cells from *n* = 6 healthy donors. The maximum dose without a change in viability was used for Bardoxolone methyl and PSTC. Both hNRF2 cmRNAs were transfected at the EC80 dose level for hNRF2 E79K cmRNA in the study to reduce confounding effects of different cmRNA concentrations and transfection reagent amounts in the study.

To confirm the specificity of the results to NRF2 protein expression rather than non-specific cmRNA-sensing, a non-coding hNRF2 STOP cmRNA was evaluated for global transcript changes. Here, all in-frame ATGs were mutated to stop codons TGA/TAG. The hNRF2 STOP cmRNA control showed an upregulation of 3–5 transcripts at each timepoint, whereas the hNRF2 cmRNAs expressed between 5 and 389 transcripts at each timepoint (Supplemental Table S3), indicating that the control only has a minor influence on the transcript levels at timepoints between 6 h and 48 h after transfection.

**Figure 3:**
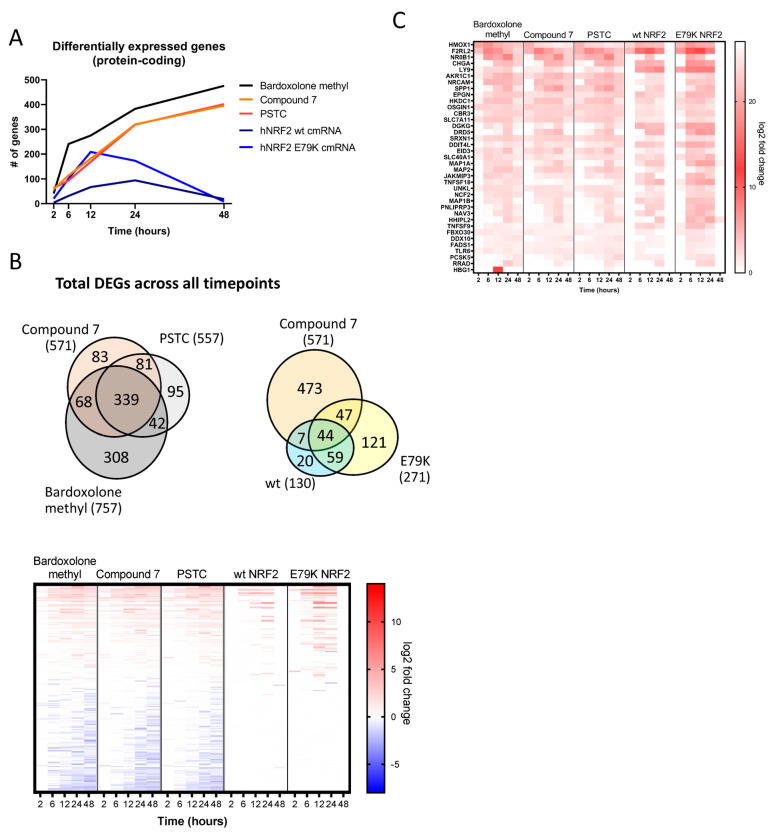
Global gene expression of hNRF2 cmRNA and small molecules in human bronchial epithelial cells: (**A**) number of differentially expressed protein-coding transcripts regulated by log2 fold-change >1 or <−1 and q < 0.05 for *n* = 4–6 donors at each timepoint. (**B**) Comparison of shared and unique differentially expressed protein-coding transcripts at any timepoint between treatment groups. (**C**) Heatmap indicating log2 fold change in all protein-coding transcripts that are regulated by all treatment groups at least 1 timepoint.

Protein-coding transcripts were selected for further analysis. Differentially expressed genes were identified by comparing Compound **7**, Bardoxolone methyl, and PSTC against a DMSO-treated control at each timepoint, while each hNRF2 cmRNA was compared against the hNRF2 STOP cmRNA. Compound **7**, Bardoxolone methyl, and PSTC treatments all resulted in a continually increasing number of differentially regulated transcripts (Figure 3A). Bardoxolone methyl resulted in the greatest number of genes regulated at the 48 h timepoint with 476 genes, as did PSTC (402 genes) and Compound **7** (396 genes). Transcriptional activity following hNRF2 wt cmRNA transfection peaked at 24 h with the maximum number of 94 regulated genes, but by 48 h, it had returned to baseline. The hNRF2 E79K cmRNA regulated a maximum of 209 transcripts at 12 h after transfection. 

Across all timepoints, Bardoxolone methyl regulated 757 transcripts in total, PSTC regulated 557, and Compound **7** regulated 571, compared to 130 transcripts from hNRF2 wt cmRNA and 271 transcripts from hNRF2 E79K cmRNA (Figure 3B). A total of 1184 genes were differentially regulated in any treatment group at at least one timepoint. A total of 37 protein-coding transcripts were regulated by all treatment groups at at least one timepoint (Figure 3C).

### 3.3. Selection of Gene Signature for Additional Studies

**Figure 4:**
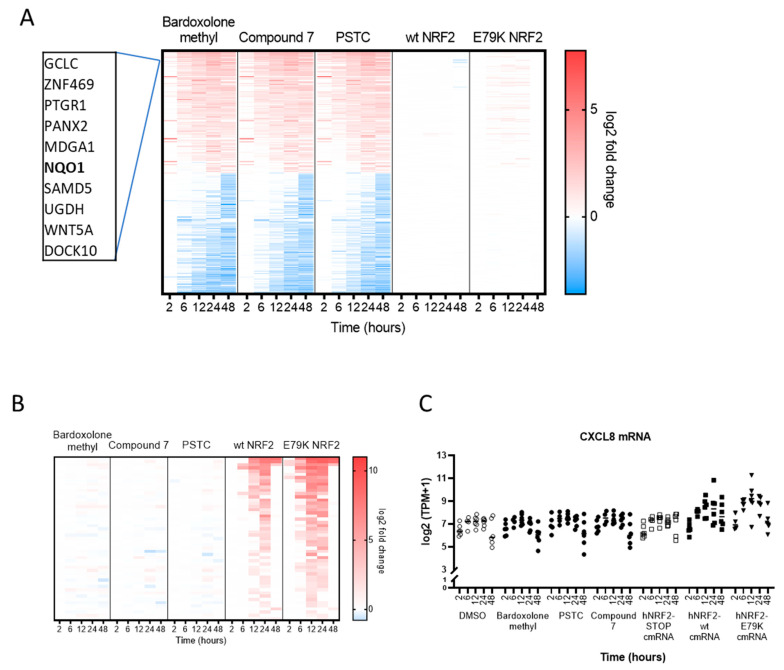

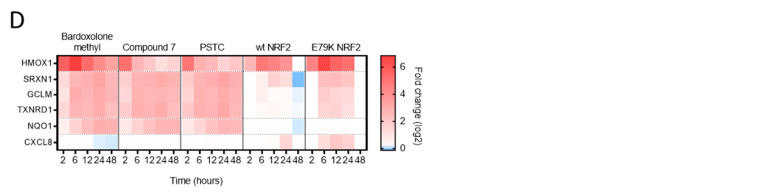
Gene signature from HBECs selected for additional profiling: (**A**) Heatmap of the protein-coding transcripts that are significantly regulated by small molecules and not significantly regulated by either hNRF2 cmRNA treatment group at any timepoint. (**B**) Heatmap of the protein-coding transcripts that are significantly regulated by hNRF2 cmRNAs and not significantly regulated by any small molecules at any timepoints. Significance measured as log2 fold-change >1 or <−1 and q < 0.05 for *n* = 4–6 donors. (**C**) Transcript per million (TPM) expression data for *CXCL8*. (**D**) heatmap of the genes selected for additional profiling.

A small gene signature was selected for follow-up studies to investigate differences in NRF2 activity in additional primary pulmonary cell types. There were 255 protein-coding transcripts regulated by the small molecules that were not regulated by either hNRF2 cmRNA (Figure 4A). Interestingly, several commonly reported NRF2 target genes were not significantly regulated following hNRF2 cmRNA transfection, of which *NQO1* was chosen for further investigation. There were 51 protein-coding transcripts differentially expressed only by hNRF2 cmRNAs and not by the small molecules (Figure 4B), all of which were upregulated. Ingenuity Pathway Analysis predicts activation of these genes is consistent with elevated cytokine signaling. One representative transcript, *CXCL8*, was selected for additional study (Figure 4C). Interleukin-8 has been previously observed with NRF2 overexpression in human cells [28]. In all, six genes were selected for further analysis: *NQO1*, *CXCL8*, *HMOX1*, sulfiredoxin 1 (*SRXN1*), thioredoxin reductase 1 (*TXNRD1*), and glutamate cysteine ligase modifier subunit (*GCLM*). These genes showed differential expression patterns across treatment groups in the HBEC transcriptomics study (Figure 4D). Compound **7** was selected as a representative small molecule for further studies since the target gene profile was similar between all small molecule compounds.

### 3.4. Expression of Functional NRF2 in Primary Human Lung Fibroblasts

**Figure 5:**
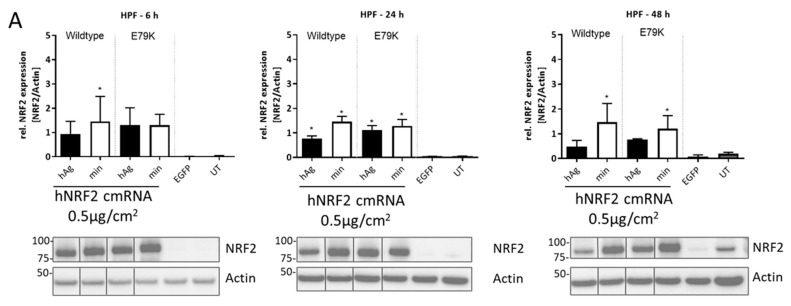

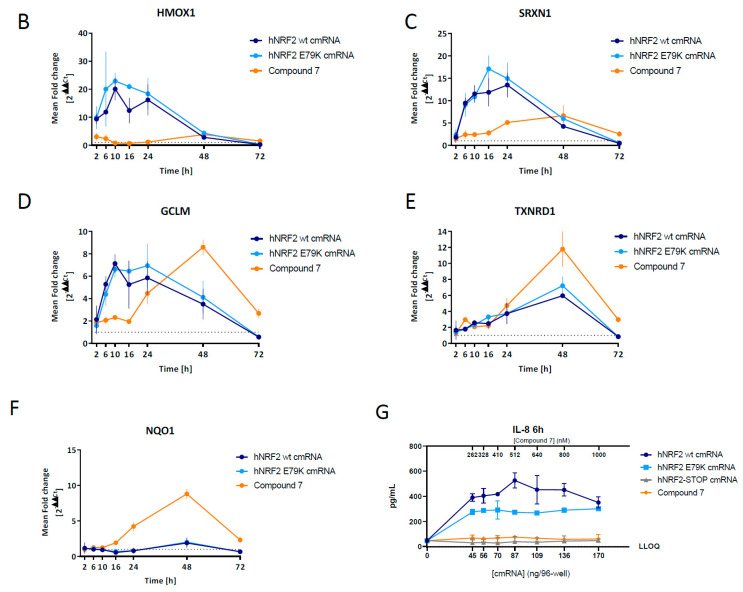
Kinetic response of human primary lung fibroblasts to NRF2 activation: (**A**) NRF2 protein expression following transfection of hNRF2 cmRNAs in HPFs. Data are pooled results from 3 independent experiments (*n* = 1 donor) showing mean ± SD. * *p* < 0.05; ordinary one-way ANOVA with Dunnett’s multiple comparisons against eGFP-transfected control at each timepoint. The full Western blot images were cropped to the relevant NRF2 band. Relative gene expression following transfection of hNRF2 cmRNAs in HPFs of (**B**) *HMOX1*, (**C**) *SRXN1*, (**D**) *GCLM*, (**E**) *TXNRD1*, (**F**) *NQO1*. Data are pooled results from 3 independent experiments showing mean ± SD. (**G**) Secreted IL-8 (pg/mL) in the supernatant of HPF cultures at 6 h after treatment. Data are pooled results from 3 independent experiments (*n* = 1 donor) showing mean ± SD.

The hNRF2 cmRNAs and Compound **7** were next profiled in primary human lung fibroblasts in a kinetic study. Expression of NRF2 protein was evaluated in human primary lung fibroblasts (HPF) following transfection of all constructs. NRF2 protein was detected above untransfected controls at all timepoints tested, beginning from 6 h and continuing through 48 h (Figure 5A).

Fibroblasts transfected with hNRF2 cmRNAs maintained *HMOX1* expression from 6 h through 24 h post-transfection (Figure 5B). Compound **7** did not induce significant *HMOX1* upregulation in the fibroblasts at any timepoint. The expression of *SRXN1* following hNRF2 cmRNA transfection was similar to *HMOX1*, with peak expression in the first 24 h (Figure 5C). Compound **7** significantly increased expression of *SRXN1* at 24 and 48 h only and did not reach the same magnitude of induction as the hNRF2 cmRNAs at 6–24 h. Upregulation of *GCLM* after hNRF2 cmRNA transfection peaked within the first 24 h, and for Compound **7** at 48 h (Figure 5D), but all treatments gave a similar magnitude fold change. Interestingly, *TXNRD1* did not reach maximum activation until 48 h after transfection with hNRF2 cmRNAs, which matched the kinetics observed with Compound **7** (Figure 5E). For *TXNRD1*, Compound **7** induced a higher maximum fold change compared to hNRF2 cmRNA transfection. Compound **7** induced significant expression of *NQO1* from 24 h, peaking at 48 h (Figure 5F). Neither hNRF2 cmRNA construct resulted in the expression of *NQO1* at the timepoints tested.

Secreted IL-8 protein was measured in the supernatant in this cell system. As NRF2 transcriptional activity was already observed at 6 h, IL-8 secretion was evaluated at this timepoint in a dose–response study. Significant IL-8 levels were detected in the supernatant at all doses in response to both hNRF2 cmRNA but not hNRF2 STOP cmRNA or Compound **7** (Figure 5G).

### 3.5. Expression of Functional NRF2 in Primary Human Alveolar Macrophages

**Figure 6:**
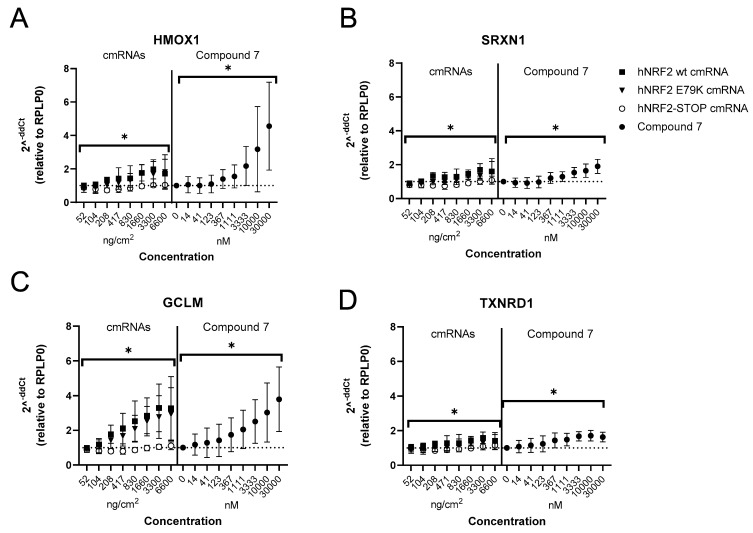

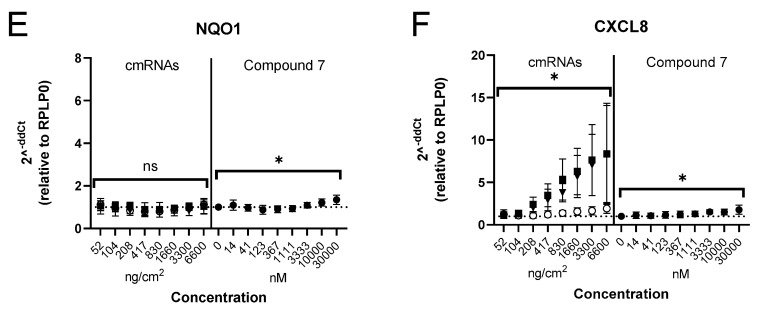
Response of human primary alveolar macrophages to NRF2 activation. Relative transcript levels in alveolar macrophages 6 h following hNRF2 cmRNA transfection or Compound **7** treatment of (**A**) *HMOX1* (**B**) *SRXN1* (**C**) *GCLM*, (**D**) *TXNRD1* (**E**) *NQO1* (**F**) *CXCL8*. Data are represented as the mean of duplicate samples in *n* = 6 donors ± SD. * *p* < 0.05 by ordinary one-way ANOVA with Sidak’s multiple comparisons (cmRNAs) or Dunnet’s multiple comparisons (Compound **7** vs. DMSO); ns: not significant.

As a final evaluation of cell-type-specific transcriptional activity of hNRF2 cmRNA and KEAP1 inhibition, the downstream effects of hNRF2 cmRNA transfection compared to Compound **7** were profiled in primary human alveolar macrophages at 6 h after treatment/transfection. Compound **7** induced significant *HMOX1* expression, while hNRF2 cmRNA transfection did not (Figure 6A). Neither *SRXN1* nor *TXNRD1* were upregulated in any treatment group (Figure 6B,D). The most significantly upregulated gene in alveolar macrophages was *GCLM*, which was upregulated by both hNRF2 cmRNAs and Compound **7** (Figure 6C). As observed in HBECs and fibroblasts, *NQO1* was not expressed following hNRF2 cmRNA transfection; however, in this cell system, it was also not induced following Compound **7** treatment (Figure 6E). The hNRF2 cmRNAs induced *CXCL8* expression, which was specific to the hNRF2-expressing constructs as neither hNRF2-STOP transfection nor Compound **7** treatment resulted in *CXCL8* up-regulation (Figure 6F).

## 4. Discussion

This study demonstrates the efficacy of cmRNA technology to overexpress human NRF2 in primary cell systems. Functional NRF2 protein was expressed from hNRF2 (wt and E79K mutant) constructs evaluated, as measured by *HMOX1* gene upregulation in all cell systems. There was no evidence of significant off-target sequence- or cmRNA-specific response, as most activity was limited to constructs that expressed functional NRF2, not the hNRF2 STOP cmRNA.

Although the E79K mutation was predicted to bypass KEAP1 interaction and thus result in a longer NRF2 half-life [27], we saw no evidence of extended activity of this cmRNA in our experiments. The only significant difference in activity observed was a higher fold change in NRF2 target gene expression.

Although the NGS study indicated that small molecule KEAP1 inhibitor treatment regulated significantly more genes over time, only a fraction of those genes were shared when NRF2 was expressed transiently from the cmRNA constructs. In follow-up studies comparing HBECs to primary pulmonary fibroblasts and alveolar macrophages, we were able to determine some consistent features of the hNRF2 cmRNA effects compared to Compound **7** across cell types. Strikingly, *NQO1* was not upregulated in any cell system by the hNRF2 cmRNAs. On the contrary, *CXCL8*/IL-8 was uniquely expressed following hNRF2 cmRNA transfection but not Compound **7** treatment. A previous report suggested that NRF2 overexpression stabilizes *CXCL8* mRNA [28].

**Figure 7:**
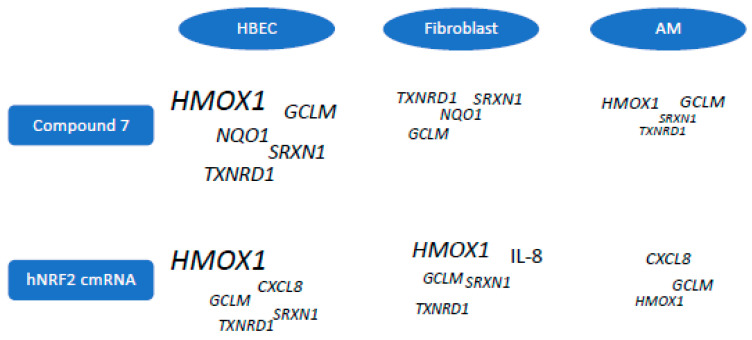
Graphical summary of gene expression preferences between primary human lung cell types to hNRF2 cmRNA and Compound **7** treatment. Preferred gene expression based on maximum log2 fold-change values in each cell system. Size of text indicates relative expression amount across all cell types.

The commonly reported NRF2 target genes (*HMOX1*, *SRXN1*, *TXNRD1*, *GCLM*) all showed cell-type-specific preferences (Figure 7). Both bronchial epithelial cell systems strongly induced *HMOX1* in response to both Compound **7** or hNRF2 cmRNAs. Alveolar macrophages responded to both stimuli with a stronger *GCLM* response than *HMOX1*. Fibroblasts strongly upregulated *HMOX1* in response to hNRF2 cmRNA transfection, but it was not detectable in response to Compound **7** at the timepoints tested.

One possible explanation for differences in target gene expression between small molecules and hNRF2 cmRNAs could be the reliance of NRF2 on cofactors for optimal gene expression. Small musculoaponeurotic fibrosarcoma proteins (MAFs) are required for gene expression of NRF2 target genes, including *NQO1* [29]. Overexpression of NRF2 above endogenous levels may overwhelm the balance between NRF2 and MAFs needed to fully activate the pathway.

There were some limitations to the present study. The transcriptomics study was performed with a relatively high dose of all the compounds, so more subtle transcriptional patterns would not be observed. Direct comparisons between the small molecule results and transfected hNRF2 cmRNAs may be biased by the fact that all cells will respond to small molecules, where only a portion of cells would be transfected with the cmRNAs. Transfection efficiency might also be different across cell systems, causing some bias in the resulting target gene expression. The fold increase in gene induction may depend on the baseline expression of the gene, which was not evaluated in this study. In addition, the selected target genes may not be representative of all genes, and further transcriptomics profiling in all cell types may provide a more complete picture. All experiments were performed on cells from healthy donors, and the results may be different in diseased tissue. In future studies, it will be interesting to compare the efficacy and safety of NRF2 overexpression versus KEAP1 inhibition in vivo to determine the translatability of the in vitro systems tested in the present study. Of significance, the relationship between NRF2 overexpression and the pro-inflammatory cytokine IL-8 will be important to understand if this targeting strategy is carried toward the clinic. Rodents lack the gene for the chemokine *CXCL8* (IL-8), and other ligands such as *Cxcl1* (Kc), *Cxcl2* (Mip2), and *Cxcl5* (Gcp2/Lix) are considered the murine functional equivalent to human *CXCL8* [30,31]. Therefore, the assessment of potential consequences of IL-8 induction will be challenging in rodents, but it could be investigated in non-rodent species as part of the non-clinical safety package during drug development.

Overall, this study provides a new data set assessing transcriptional changes in multiple primary lung cell systems after small molecule inhibition of the KEAP1-NRF2 interaction versus overexpression of NRF2. This work provides evidence that the NRF2 gene signature depends highly upon the cell system and mode of activation of NRF2 regulation and begins to establish these differences in multiple lineages of primary human lung cells. Additional studies in the future will be important to uncover the mechanisms that underlie each cell system’s unique response to different stimuli.

## Figures and Tables

**Table 1:** Transfection conditions for alveolar macrophages.

Plate Format	RNA [µg]/Well	Transfection Reagent [µL]/Well	Lipoplexes/Well
96-well	2 µg–2.7 ng (1:3 dilution series in 5 µL Opti-MEM)	0.3 µL in 5 µL Opti-MEM	10 µL

## Data Availability

Data for this manuscript are available in the main text or Supplementary Materials. RNA-sequencing data underlying the findings described in this manuscript may be obtained in accordance with AstraZeneca’s data-sharing policy described at https://astrazenecagrouptrials.pharmacm.com/ST/Submission/Disclosure, accessed 20 September 2023. Use the “Enquiries about Vivli Member Studies” (https://vivli.org/members/enquiries-about-studies-not-listed-on-the-vivli-platform/) form and include the publication title and data accession number GSF1488284 in your request.

## Supplementary Materials

The following supporting information can be downloaded at: https://www.mdpi.com/article/10.3390/antiox13080924/s1, Supplemental Figure S1. Selection of doses for NGS study in HBECs. Supplemental Table S1. List of Roche probes for RT-qPCR in BEAS2B and HPF cell systems. Supplemental Table S2. List of Taqman assays for RT-qPCR in HBEC and macrophage cell systems. Supplemental Table S3. Differentially expressed genes in cmRNA-transfected cells compared to untreated cells.
