# Supplementary material for: Transcriptional Dynamics of NRF2 Overexpression and KEAP1-NRF2 Inhibitors in Human Cell Line and Primary Lung Cells
Source: Antioxidants (Basel). 2024 Jul 30;13(8):924. doi: 10.3390/antiox13080924 (PMC11351141; doi:10.3390/antiox13080924)
Supplement: Supplementary file 1 [file antioxidants-13-00924-s001.zip › antioxidants-3092072-supplementary.pdf]

Supplemental information

Methods

Human bronchial epithelial cells (HBECs) dose-response

Culture and transfection in Methods section. At the end of the experiment, cells were washed once in PBS and lysed in 150 µL of Qiagen RLT lysis Plus buffer (Qiagen Hilden, Germany). Lysates were immediately stored at -80 deg C until RNA purification with RNeasy 96 Plus kit (Qiagen). cDNA was prepared with High Capacity cDNA Reverse Transcription kit (Applied Biosystems, Waltham Massachussetts). qPCR was performed with TaqMan Fast Advanced Mastermix (Applied Biosystems) on a QuantStudio 7 Flex Real-Time PCR system (Applied Biosystems), using the and primer-probe sets in Supplemental Table S2. Data was analyzed using automatic threshold and baseline using the platform software and analysed using GraphPad Prism statistical software.

Off-target differential gene expression analysis in NGS study

Methods were performed as in main text. An adjusted p-value of <0.05 was considered significant with a log2foldchange cut-off of 0.5.

Supplemental Table S1. List of Roche probes for RT-qPCR in BEAS2B and HPF cell systems

**Target Gene**<br>**Gene accession number**	**Sequence Forward**	**Sequence Reverse**	**UPL**
ActB<br>NM_001101.5	AGAGCTACGAGCTGCCTGAC	CGTGGATGCCACAGGACT	#9

RPLP0<br><br>NM_001002.3	TCTACAACCCTGAAGTGCTTGAT	CAATCTGCAGACAGACACTGG	#6
HPRT1<br><br>NM_000194.3	TGACCTTGATTTATTTTGCATACC	CGAGCAAGACGTTCAGTCCT	#73
HMOX1<br><br>NM_002133.2	AGACTGCGTTCCTGCTCAAC	GGCTCTGGTCCTTGGTGTC	#17
SRXN1<br><br>NM_080725.2	CAGTGCTCGTTACTTCATGGTC	CCTTCCTTGAACGCAGACAT	#46
TXRND1<br><br>NM_001093771.2	TGTGGACTGACCAAAAAGCA	GCGCTTGGTCACAGACAAT	#56
NQO1<br><br>NM_000903.2	CGGCTTTGAAGAAGAAAGGAT	CGCAGGGTCCTTCAGTTTAC	#22
GCLM<br><br>NM_001308253.1	GACAAAACACAGTTGGAACAGC	CAGTCAAATCTGGTGGCATC	#18

Supplemental Table S2. List of Taqman assays for RT-qPCR in HBEC and macrophage cell systems

**Gene**	**Human**	**Gene Accession Number**
HMOX1	Hs01110250_m1	NM_002133.2
SRXN1	Hs00607800_m1	NM_080725.2
GCLM	Hs00978072_m1	NM_001308253.1
TXNRD1	Hs00917067_m1	NM_001093771.2
NQO1	Hs01045993_g1	NM_000903.2
RPLP0 (HBECs)	Hs99999902_m1	NM_001002.3
RPLP0 (Macrophages)	Hs00420895_gH	NM_001002.3
GAPDH	Hs99999905_m1	NM_001289746.1
TNFa	hs00174128_m1	NM_000594.3

CXCL8	hs00174103_m1	NM_000584.3

Supplemental Table S3. Differentially expressed genes in cmRNA-transfected cells compared to untreated cells.

The number of transcripts with a gene symbol that are differentially regulated be hNRF2 constructs relative to untreated cells in n=4-6 healthy donors.

**DEGs**	**2h**	**6h**	**12h**	**24h**	**48h**
hNRF2 STOP cmRNA vs untreated	4	5	5	3	4
hNRF2 wt cmRNA vs untreated	5	37	93	145	77
hNRF2 E79K cmRNA vs untreated	29	188	389	286	40

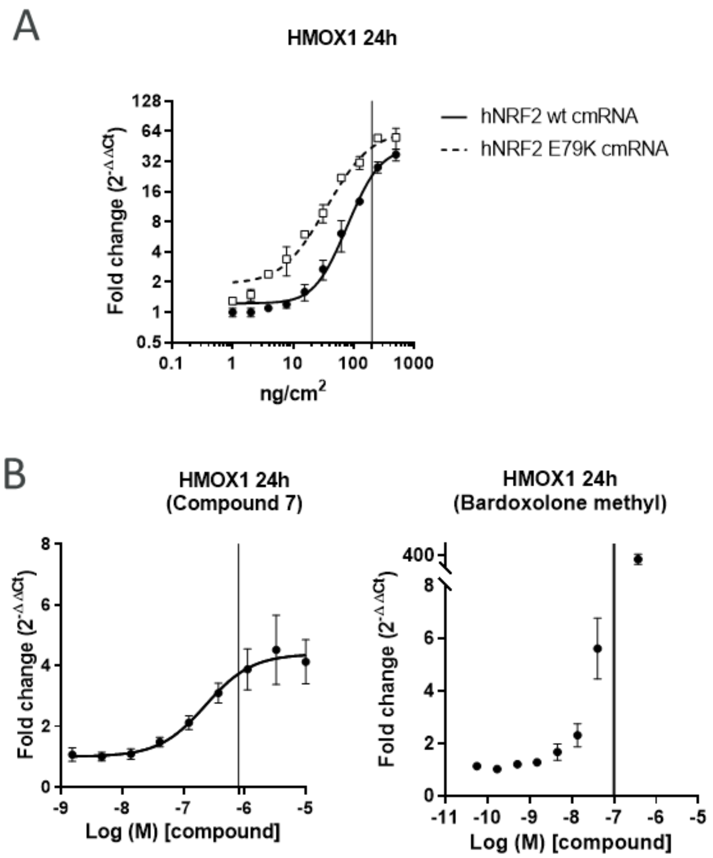

Supplemental Figure S1. Selection of doses for NGS study in HBECs.

Dose reponse curves for each treatment on HMOX1 gene expression at 24 hours after treatment start. Data represents values from n=3 donors shown as mean +/- SD. Vertical line indicates the dose chosen for the NGS study.
